# Enhancing Automated Brain Tumor Detection Accuracy Using Artificial Intelligence Approaches for Healthcare Environments

**DOI:** 10.3390/bioengineering11060627

**Published:** 2024-06-19

**Authors:** Akmalbek Abdusalomov, Mekhriddin Rakhimov, Jakhongir Karimberdiyev, Guzal Belalova, Young Im Cho

**Affiliations:** 1 Department of Computer Engineering, Gachon University, Sujeong-gu, Seongnam-si 13120, Gyeonggi-do, Republic of Korea; akmaljon@gachon.ac.kr; 2 Department of Artificial Intelligence, Tashkent University of Information Technologies Named after Muhammad Al-Khwarizmi, Tashkent 100200, Uzbekistan; raximov022@gmail.com (M.R.); jahongirkarimberdiyev618@gmail.com (J.K.); 3 Department of Information Systems and Technologies, Tashkent State University of Economics, Tashkent 100066, Uzbekistan; g.belalova@tsue.uz

**Keywords:** YOLOv5, non-local neural networks, brain tumor, MRI, segmentation, object detection, deep learning

## Abstract

Medical imaging and deep learning models are essential to the early identification and diagnosis of brain cancers, facilitating timely intervention and improving patient outcomes. This research paper investigates the integration of YOLOv5, a state-of-the-art object detection framework, with non-local neural networks (NLNNs) to improve brain tumor detection’s robustness and accuracy. This study begins by curating a comprehensive dataset comprising brain MRI scans from various sources. To facilitate effective fusion, the YOLOv5 and NLNNs, K-means+, and spatial pyramid pooling fast+ (SPPF+) modules are integrated within a unified framework. The brain tumor dataset is used to refine the YOLOv5 model through the application of transfer learning techniques, adapting it specifically to the task of tumor detection. The results indicate that the combination of YOLOv5 and other modules results in enhanced detection capabilities in comparison to the utilization of YOLOv5 exclusively, proving recall rates of 86% and 83% respectively. Moreover, the research explores the interpretability aspect of the combined model. By visualizing the attention maps generated by the NLNNs module, the regions of interest associated with tumor presence are highlighted, aiding in the understanding and validation of the decision-making procedure of the methodology. Additionally, the impact of hyperparameters, such as NLNNs kernel size, fusion strategy, and training data augmentation, is investigated to optimize the performance of the combined model.

## 1. Introduction

Brain tumors represent a considerable global health issue, presenting a significant danger to individuals across various age groups [1]. These anomalous cell proliferations within the brain have the potential to result in severe health implications and, in certain instances, can lead to fatality. Timely identification and precise diagnosis of brain tumors play a pivotal role in ensuring effective therapeutic interventions and better prognoses for patients [2]. Recent progress in medical imaging tools and computational methodologies has facilitated the emergence of computer-assisted detection systems designed to aid healthcare practitioners in the recognition and precise localization of brain cancers [3].

Conventional approaches to the identification of brain malignancies and gliomas traditionally depended on the manual interpretation of medical imaging modalities, notably magnetic resonance imaging (MRI) and computed tomography (CT) scans [4]. Nonetheless, the subjective nature inherent in visual analysis and the intricacies associated with tumor identification frequently presented challenges for radiologists, thereby contributing to the risk of misdiagnosis or delayed commencement of treatment. Consequently, a heightened interest has emerged in harnessing the capabilities of artificial intelligence (AI) and machine learning techniques to augment the precision and efficiency of brain tumor detection [5].

AI has exhibited remarkable promise across diverse domains, encompassing the examination of medical imaging. Deep learning (DL) frameworks, similar to convolutional neural networks or other AI approaches, have showcased exceptional abilities in discerning patterns, extracting features, and executing classification tasks. Leveraging these models, particularly in processing extensive datasets of medical images, enables efficient learning of intricate representations, empowering them to differentiate between healthy brain tissue and areas affected by tumors [6].

Transfer learning, a DL technique, has gained significance in the realm of medical imaging [7]. This method capitalizes on pre-trained models using extensive datasets, enabling researchers to commence their models with acquired features and tailor them to particular tasks using smaller datasets. This approach not only expedites the training phase but also amplifies the models’ capacity for generalization and performance.

Deep learning methods have made impressive progress in a variety of computer vision applications in recent years, most notably on the object detection area. Among these models, YOLOv5 (You Only Look Once) has garnered considerable acclaim owing to its speed and precision in object detection applications. Our objective revolves around harnessing the capabilities of the YOLOv5 model to investigate its potential in accurately and efficiently detecting brain tumors within MRI scans [8].

Beyond the utilization of the YOLOv5 model, our approach incorporates non-local neural networks (NLNNs) to augment the efficacy of brain tumor detection [9]. NLNNs, belonging to a category of deep neural networks, excel in grasping extensive contextual connections within images, facilitating the extraction of contextual information and enhancing predictive accuracy. Introducing NLNNs into our detection framework aims to harness their capacity in modeling spatial correlations and capturing comprehensive global context. This integration proves particularly advantageous in discerning nuanced tumor characteristics and effectively distinguishing them from healthy brain tissue.

The fusion of YOLOv5 and NLNNs presents a promising avenue for achieving heightened accuracy and resilience in brain tumor detection [10]. The YOLOv5 model’s adeptness in precise object detection and localization, coupled with NLNNs’ proficiency in capturing intricate details and contextual nuances, stands to bolster overall detection performance. This research endeavors to scrutinize the efficacy of this amalgamated methodology and appraise its capability to elevate the precision in identifying brain tumors [11].

This study aims to construct a deep learning framework by integrating YOLOv5 and NLNNs for the detection of brain tumors within MRI scans. The primary objectives involve the development and evaluation of this framework using an extensive dataset containing annotated brain MRI images. The methodology includes training and refining the YOLOv5 model on the dataset while leveraging NLNNs to augment its functionalities [12]. The assessment will encompass an evaluation of detection accuracy, precision, and computational efficiency of the proposed framework, followed by a comparative analysis against established methods in the field.

This research makes significant contributions by conducting a comprehensive examination of the YOLOv5 model and NLNNs concerning their efficacy in brain tumor detection. Through rigorous evaluation on a sizable dataset, this study offers insights into the amalgamation of these models, showcasing their potential to elevate detection accuracy [13]. The implications of these findings are poised to propel advancements in brain tumor detection, potentially guiding the development of automated systems capable of aiding medical professionals in more efficient brain tumor diagnoses.

Our proposal introduces a brain tumor detection system built upon an improved YOLOv5 model [14] designed to address the aforementioned limitations. For tumor identification within MRI images, a foundational framework pre-trained on the common objects in context (COCO) dataset was utilized. Pre-trained weights were used as the backbone network’s initialization parameters in order to optimize the network structure parameters and strengthen the original network. This study drew inspiration from our prior research outcomes [15]. Section 3 and Section 4 delineate our efforts to enhance the performance of the conventional YOLOv5 network, facilitating swift brain tumor detection, with subsequent validation on AI mainframes.

The main achievements of this study involve creating an automated system for detecting brain tumors with reduced false-positive results. A substantial brain tumor MRI dataset was curated, significantly enhancing the precision of the deep convolutional neural network model. Refinements in anchor-box clustering via the K-means+ technique were implemented to mitigate misclassifications. The optimization of the spatial pyramid pooling fast (SPPF) layer within the backbone aimed to specifically target smaller features, while adjustments in the neck part utilizing the bidirectional feature pyramid network (Bi-FPN) module were executed to ensure effective fusion of multi-scale features. Lastly, improvements in the system design, accurate detection rate, and speed were achieved through the application of network pruning and transfer learning methodologies during training.

The following sections of this manuscript are organized in the following manner: Section 2 furnishes a comprehensive review of pertinent literature about the identification of brain cancer and the utilization of AI methodologies. Section 3 expounds on the methodology employed, encompassing details on the YOLOv5 model, NLNNs, and the dataset under consideration for this study. Section 4 delineates the outcomes of our experiments and provides an assessment of the system’s performance. In conclusion, Section 5 summarizes key findings, draws conclusions, and delineates potential avenues for future research in this domain.

## 2. Related Works

Combining several imaging methods with deep learning (DL) models has significantly advanced computer-aided diagnosis (CAD) systems for distinguishing brain malignancies that are pituitary, meningioma, and glioma. Cheng et al. conducted a noteworthy study leveraging Content-based Image Retrieval (CBIR) techniques alongside a powerful database comprising 3064 T1-weighted contrast-enhanced (CE) MRI pictures for extracting brain tumors. Their innovative framework incorporated adaptive spatial division, segmenting tumor regions into subregions based on intensities. Utilizing the Fisher mask to amalgamate these areas and generate a picture-level signature resulted in an impressive Mean Average Precision (mAP) of 94.68%. This research has spurred further exploration into the potential of DL methods for the categorization of these three kinds of MRI-diagnosed brain tumors [14].

In a notable research endeavor by Swati et al., a pre-trained deep convolutional neural network (DCNN) named VGG19 was employed, utilizing transfer learning to harness crucial characteristics for image identification [15]. Through fine-tuning the VGG19 model, accurate classification of brain tumor images was accomplished, reaching a 94.82% categorization correctness. Likewise, Deepak et al. adopted transfer learning with another pre-trained DCNN, GoogleNet, implementing a patient-level five-fold cross-validation approach. Their research yielded an impressive accuracy of 98% in classifying the three distinct brain tumors [16].

Rehman et al. utilized computer vision methodologies to magnify their database, thereby enhancing the efficacy of their model [17]. Employing diverse affine transformations on image samples facilitated the extraction of supplementary features by their chosen DCNN models (specifically, AlexNet, GoogleNet, and VGG16). Researcher’s categorizer demonstrated predictions rate of 96.98%, 97.76%, and 97.14%, accordingly. In a separate study, Sultan et al. introduced a tailored CNN model designed for the multi-class classification of brain tumors. This architecture incorporated activation functions, normalization techniques, pooling layers, and dropout mechanisms to counter overfitting. Remarkably, exceeding current state-of-the-art approaches, their model attained an outstanding accuracy rate of 97.7% [18].

Noreen et al. recently introduced more advanced DCNN models, DenseNet201 and InceptionV3, in a study focused on brain tumor diagnosis [19]. Their methodology involved a concatenated multi-stage feature extraction process tailored for tumor analysis, resulting in remarkable accuracies of 99.34% for InceptionV3 and 99.51% for DenseNet201. In a related context, Bhanothu et al. utilized the Faster R-CNN object detection approach to identify brain tumor locations within MRI scans, utilizing bounding boxes for identification purposes [20]. Despite the promise of DCNN item recognition model, Faster R-CNN encountered hurdles at this nascent stage and achieved a modest mean average precision (mAP) of only 77.60%.

The studies discussed above collectively showcase substantial progress in brain tumor diagnosis, particularly in the realms of classification and manually crafted segmentation techniques [21]. Although the evolution of object detection methodologies within DCNNs is ongoing, recent strides highlight encouraging prospects for enhanced outcomes in this domain (Table 1).

Al-Masni conducted a study utilizing the “You Only Look Once” (YOLO) detection model to simultaneously identify breast masses across multiple digital mammograms. Diverging from algorithms targeting specific patches or regions, YOLO examined the entire input image throughout both testing and training stages. That strategy conferred a notable velocity benefit and diminished overhead in contrast to region-centric techniques such as Fast R-CNN and the conventional window-sliding method. Their investigation highlighted YOLO’s capacity to augment computer-aided diagnosis (CAD) by reliably detecting masses with a precision rate of 98.8%.

Ünver et al. supervised a study underscoring the efficacy of YOLO in the realm of medical imaging [22]. Employing a YOLOv3 model trained with 2000 annotated images, they focused on identifying skin lesions without employing any augmentation techniques. Through the integration of the GrabCut segmentation algorithm, their approach attained an accuracy rate of 93.39% when validated against 500 images, surpassing the performance of alternative models like ResNet and U-Net. The findings of the study emphasize the potential of YOLO to significantly contribute to tackling diverse challenges within medical imaging.

Previously, YOLO models encountered challenges due to high computational demands and moderate performance, limiting their viability for future applications and deployment. Consequently, the use of them in medical imaging was the subject of very little research. However, recent strides in YOLOv3 and the introduction of YOLOv4 have significantly enhanced object detection capabilities compared to alternative solutions, all while maintaining lower resource requirements. Therefore, this research aims to evaluate how well the modified YOLOv4 model performs in terms of training an automated CAD recognition technique that is specifically designed to identify brain malignancies of the pituitary, glioma, and meningioma.

The objective is to aid medical professionals in the diagnostic process by utilizing a streamlined YOLOv4 model, consuming fewer computational resources and disk space. This feature facilitates its deployment across diverse platforms. Considering insights from prior studies employing YOLO in medical imaging, there remains potential for refinement in automating the detection of MRI scans showing brain malignancies. As of the present time, no studies have utilized a finely tuned YOLO-based model employing transfer learning model for MRI brain cancer identification. This underscores the necessity to assess its overall precision, indicating an unexplored avenue for enhancing automated brain tumor detection in medical imaging.

When applying AI techniques to improve the accuracy of automated brain tumor diagnosis in healthcare settings, several technical shortcomings frequently call for the development of better procedures. The primary technical deficiencies that frequently occur in this field are listed below, and they inspired the design of the suggested technique. Limited dataset size and diversity are the main issues of all healthcare research such as brain tumor detection. Due to their limited size and lack of diversity, many of the datasets currently in use for training AI models might cause overfitting and poor generalization to new data. To increase the robustness and generalizability of the AI models, data augmentation techniques and the aggregation of larger, more diverse datasets from numerous sources are used. In addition, inconsistent data quality, insufficient model interpretability, class imbalance in datasets, and limited integration with clinical workflows are also key technical gaps that commonly arise in this domain.

## 3. Materials and Methods

### 3.1. The Proposed Model for Detecting Brain Tumors in MRI Scans

Detecting brain tumors within medical images poses a formidable challenge due to the diverse characteristics present in tumors, including variations in size, shape, and location [23]. Numerous methodologies have emerged to tackle this challenge, each with distinct strengths and limitations. However, to fairly evaluate these methods, the presence of a benchmark dataset becomes imperative, allowing for the comprehensive assessment of their efficacy [24].

Moreover, brain tumor images exhibit varying quality, influenced by factors such as sharpness, contrast, the number of slices, and pixel spacing. In our paper, we introduce the technical intricacies and architectural framework of our proposed system, specifically designed to facilitate swift and precise detection of brain tumors in medical images. This system aims to address the complexities posed by diverse tumor characteristics and image quality variations, enabling efficient and accurate tumor detection within this critical medical domain.

Past investigations have explored diverse methodologies for the detection and characterization of brain tumors, but the successful application of these approaches has been limited to a select number of studies, resulting in inconsistent outcomes. Our primary objective is to achieve precise detection of brain tumors in MRI scans. Following a comprehensive evaluation of various models, we opted for the YOLOv5 model, known for its effectiveness in detecting brain tumors.

However, faced with the challenge of insufficient training data, we initially pre-trained the YOLOv5 model using the COCO dataset to acquire foundational image recognition features. Nevertheless, the transfer of pre-trained features and hyperparameter settings from COCO presented challenges in accurately identifying brain tumors in MRI images. Consequently, we undertook a process of fine-tuning and refinement of the model, specifically tailoring it for the nuanced problem of identifying brain tumors. This iterative refinement aims to improve the functioning of the model and ensure its suitability to explain the subtleties of using MRI scans to diagnose brain tumors.

In pursuit of our goal, we initiated the YOLOv5 model with weights derived from the COCO dataset and proceeded to conduct additional training using a specifically labeled dataset comprising MRI brain tumor images. This training process entailed the refinement of deep learning algorithms, adjustment of hyperparameters, and implementation of transfer learning techniques [25]. The overarching objective was to minimize overall loss and maximize accuracy, necessitating the careful selection of an appropriate optimizer to fine-tune the neural network’s biases and learning rate.

The choice of optimizer is pivotal in ensuring the effective training of the model. In this study, researchers employed the binary cross-entropy loss function coupled with the Adam optimizer [26]. This combination was chosen to facilitate the optimization process, aiming to strike a balance between minimizing the loss function and enhancing the accuracy of the YOLOv5 model for the specific task of brain tumor.

As a result of these endeavors, we successfully crafted a YOLOv5 model with the capacity to accurately discern MRI frames featuring meningioma, glioma, and pituitary tumors. The integration of fine-tuning and transfer learning techniques significantly bolstered the model’s proficiency in identifying brain cancers, leading to enhanced accuracy. The optimized model has demonstrated promising outcomes in the realm of brain tumor detection tasks, as documented in our study [27].

Brain tumors represent abnormal cell growth within the brain, classified as either benign (noncancerous) or malignant (cancerous) formations [28]. Primary brain tumors originate within the brain itself, while secondary brain tumors manifest when cancerous cells metastasize to the brain from other bodily regions. There are many different kinds of primary brain tumors, including meningiomas, gliomas, craniopharyngiomas, pituitary adenomas, medulloblastomas, and germ cell tumors [29]. Each subtype displays unique characteristics in terms of growth patterns, affected brain areas, and clinical implications.

### 3.2. Data Preparation

The dataset employed in this study was acquired from Cheng et al., who gathered MRI scans from Nanfang Hospital in Guangzhou, China, spanning the years 2005 to 2010 [30]. The dataset’s particulars, delineated in Table 2, include specifications such as the inclusion of 2D slices of T1-weighted contrast-enhanced MRI images. This dataset encompasses a collective count of 708 instances of meningioma, 1426 instances of glioma, and 930 instances of pituitary brain tumors.

**Figure 1:**
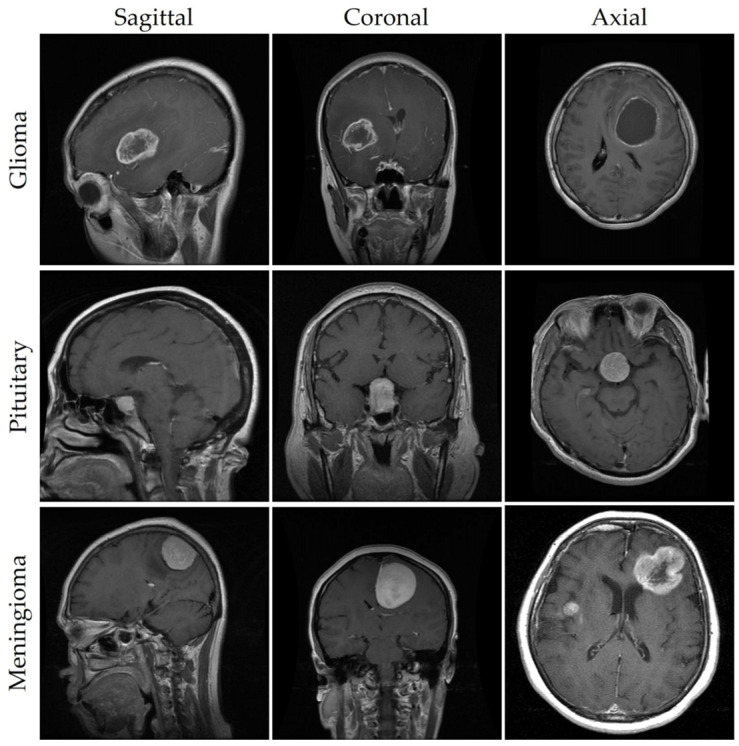
MRIs of brain tumors captured from different perspectives.

Figure 1 shows a curated selection of samples from each class in the dataset, featuring diverse perspectives such as axial, coronal, and sagittal views. According to the primary data source, these samples were taken from 233 anonymized patients and carefully curated and validated by a skilled radiologist. The images presented in the table adhere to a standard dimension scale of 512 × 512 and possess a pixel size of 49 × 49 mm. Initially stored in the MAT format, these frames underwent image processing techniques for requisite adjustments. To ensure compatibility with the YOLO model and facilitate accessibility, all images were subsequently converted to the JPG format, representing them as 2D arrays. Furthermore, in order to get rid of any potential inconsistencies during further model testing, pixel intensities were normalized using the min–max method.

In order to streamline the training and testing procedures, the comprehensive dataset underwent a stratified division into two subsets: a training set and a testing set [31]. This partitioning was executed based on a specific MRI view and class, as delineated in Table 3. The rationale behind this approach was to evaluate the model’s efficacy in accurately detecting tumors within the test data, which constituted 20% of the entire dataset. The remaining 80% of the dataset was allocated to furnish a sufficiently large pool of learnable patterns for training the models.

Furthermore, to uphold impartiality and eliminate potential biases, the selection of samples for both sets adhered to a random and unbiased methodology, devoid of any predetermined choices. This procedural rigor aimed to forestall any unwarranted outcomes or preconceived results, thus contributing to the overall reliability of this study.

The database underwent partitioning into distinct training and testing sets based on MRI view and class, facilitating impartial model assessment. Consequently, the model’s effectiveness can be evaluated on previously unseen data, owing to this segregation. This approach serves to gauge methods’ adaptability and proficiency in brain cancer detection by subjecting them to testing on data not utilized during training. The selection of testing set samples is conducted impartially through stochastic collection, thereby mitigating any potential biases or selection biases. Such measures preclude the introduction of biases that could skew evaluation outcomes in favor of specific models or assumptions.

### 3.3. Data Preprocessing

**Figure 2:**
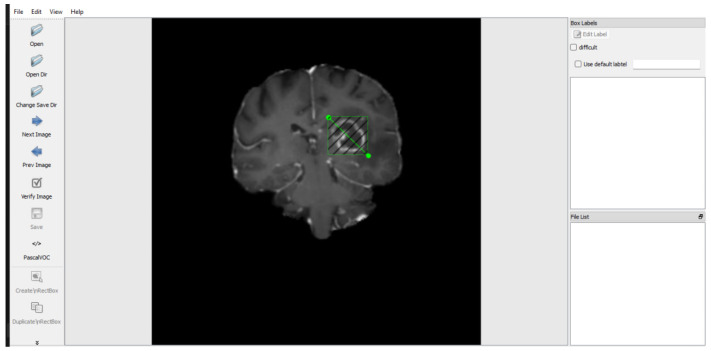
The preprocessing of the brain tumor images dataset [33].

In the analysis and detection of brain tumors, preprocessing of brain cancer pictures stands as a pivotal phase [32]. This section delineates the preprocessing techniques employed on the brain tumor image dataset to ensure the attainment of precise and dependable outcomes. Figure 2 visually represents the manual labeling process carried out on the images within the dataset. The figure illustrates the sequential steps involved in manually annotating and labeling the images, highlighting the meticulous method of precisely locating and labeling each image’s regions of interest. This step ensured that subsequent analysis focused specifically on the tumor and its surrounding structures [33].

The following steps were performed to prepare the images for subsequent analysis: image rescaling was done to establish consistency and facilitate efficient processing, where the brain tumor frames were rescaled to a standardized size of 256 × 256 pixels. This resizing step ensured that all images have the same dimensions, regardless of their original resolution [34].

Intensity normalization was employed to standardize the image intensities across different scans [35]. This step reduces variations caused by different imaging protocols or equipment. Min–max scaling was applied to map the intensity values to a normalized range, or alternatively, z-score normalization was used to transform the intensities to have a mean of zero and a standard deviation of one.

Noise reduction techniques were applied to improve image quality and minimize the impact of noise on subsequent analysis [36]. Gaussian smoothing and median filtering were utilized to reduce noise artifacts in the brain tumor images. These techniques effectively suppressed high-frequency noise while preserving important image details [37].

Skull stripping was performed to focus the analysis solely on the brain tissue and remove extraneous regions, such as the skull and non-brain structures [38]. A combination of automated and manual techniques, including region growing and thresholding, was employed to segment and extract the brain region from the images. This step ensured that subsequent analysis was focused on the relevant brain structures.

Image registration was performed to align brain tumor images to a common coordinate system, accounting for variations in patient positioning and imaging protocols. Rigid or deformable registration algorithms were employed to align the images, enabling accurate comparisons between different scans or time points [39].

Bias field correction techniques were applied, as imperfections in the imaging system can cause intensity variations across the brain tumor images. Bias field correction techniques, such as N4ITK or FSL FAST, were applied to remove these intensity variations [40]. This step ensured that the images were free from intensity biases, providing a more accurate representation of the underlying tissue structures [41].

Image enhancement techniques were applied to improve visualization and highlight subtle image features. Histogram equalization, adaptive contrast enhancement, and CLAHE were employed to enhance the image contrast while preserving important details. These techniques improved the visibility of tumor regions and facilitated subsequent analysis [42].

With region of interest extraction, the identification of the region of interest (ROI), which includes the tumor and surrounding brain tissues, was a critical step in the analysis of brain cancer pictures [43]. Manual or automated segmentation techniques, such as thresholding, active contours (e.g., level sets), or deep-learning-based segmentation models, were utilized to extract the ROI accurately.

Through the application of these preprocessing approaches to the brain tumor image dataset, several enhancements were achieved. Standardization of the images occurred, reducing noise while simultaneously amplifying critical features [44]. This comprehensive preprocessing pipeline ensured the dataset was suitably primed for subsequent analysis, significantly aiding in the precise identification and characterization of brain tumors.

### 3.4. The Architecture of YOLOv5

The YOLOv5 object detection framework, known for its real-time, one-stage capabilities, stands out as an appropriate option for our requirements due to its fast inference speed and superior object identification accuracy [45]. Thanks to the continued work of its developers, YOLO has established itself as a highly effective solution for object detection across Pascal VOC as well as the Microsoft COCO datasets. The four main variants of YOLOv5 are the benchmark YOLOv5l, the extended YOLOv5x, and the simplified preset versions, YOLOv5s and YOLOv5m. The number of feature extraction modules and convolution kernels installed at various network nodes, which leads to decreased overall model sizes and parameter counts, is the main difference between these network types.

**Figure 3:**
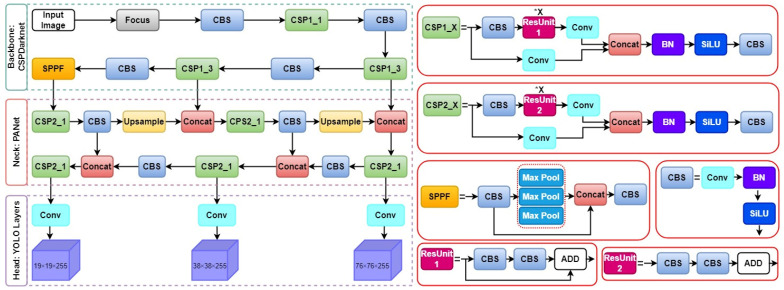
The overall architecture of the YOLOv5 model.

Figure 3 provides an overview of the comprehensive network architecture of the YOLOv5 system. The YOLOv5 model is composed of three fundamental components: the backbone, neck, and head. Initially, Cross-Stage-Partial (CSP) 1 and CSP2, featuring two distinct bottleneck CSP structures, aimed at reducing redundant information. This scaling down of floating-point operations per second (FLOPS) and model parameters has a dual impact, expediting the inference process and concurrently enhancing precision, leading to a more compact model size. Specifically, CSP1, designated as the backbone, and CSP2, referred to as the neck, were utilized for feature fusion, as elaborated below.

Furthermore, along with CSP1, the core architecture integrates Convolution Layer + Batch Normalization + Sigmoid Linear Unit (CBS) and spatial pyramid pooling fast modules. The spatial pyramid pooling fast module comprises three consecutive 5 × 5 MaxPool layers, processing input sequentially through each layer and then combining the outputs using a Concat operation, followed by a CBS operation. This method of spatial pyramid pooling, known as spatial pyramid pooling fast, is recognized for its efficiency, delivering comparable results more rapidly compared to traditional spatial pyramid pooling techniques.

Lastly, the Neck component incorporates a PANet, which utilizes an improved bottom-up pathway structure. PANet integrates a novel Feature Pyramid Network (FPN) to convey feature information efficiently starting from the lowest feasible level.

### 3.5. Non-Local Neural Networks

The structure of non-local neural networks (NLNNs) is characterized by specialized layers or modules designed to facilitate the modeling of extensive dependencies and the incorporation of global context within an image [46]. These dedicated layers augment the capacity of neural networks to extract meaningful features by taking into account the interdependencies among distant regions.

**Figure 4:**
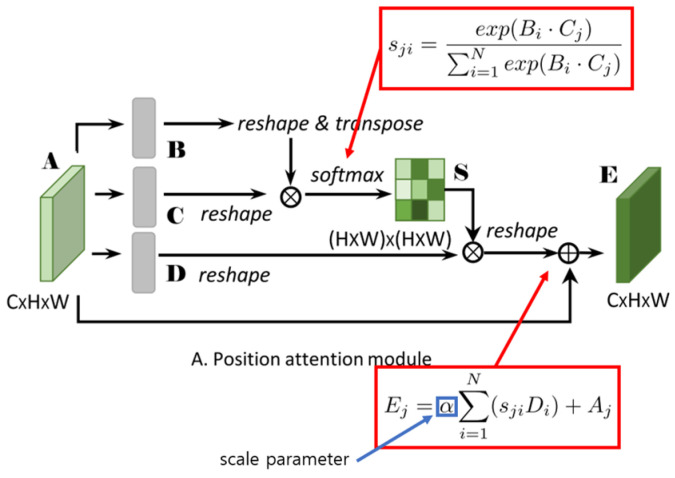
The overall architecture of the NLNNs model.

The fundamental element of NLNNs is the non-local operation, pivotal for capturing long-range dependencies [47]. This operation calculates the response at a specific position by consolidating information from all positions in the input feature map. It accomplishes this through a two-step process: first, a pairwise similarity computation is performed between the query position and all other positions, and second, a weighted sum of the values at those positions is computed. The resulting response signifies the contribution of each position to the query position, taking into account their spatial relationships. The architecture of NLNNs is depicted in Figure 4 [48].

The overarching architecture of NLNNs can be succinctly summarized as follows.

The input to an NLNN typically comprises a feature map or tensor extracted from an image through prior layers of a neural network. This feature map contains local features that NLNNs aim to improve by capturing non-local dependencies.

The pivotal element of NLNNs, the non-local operation, processes the input feature map, computing non-local responses by evaluating pairwise similarities between positions and aggregating information accordingly. The resulting response map encompasses enhanced features that effectively capture long-range dependencies.

The non-local responses are typically merged with the original input feature map through an integration step [49]. This integration may involve element-wise addition, concatenation, or other operations designed to fuse local and non-local information.

NLNNs may incorporate extra layers or modules to further process the enhanced feature map. These can include convolutional layers, pooling layers, or other components commonly found in neural network architectures [50]. The configuration of these layers is contingent upon the overall design of the NLNN and the specific task.

The final output of the NLNN is achieved by running the processed feature map through the remaining layers of the network. These layers can include classification layers, regression layers, or other components tailored for the specific task being performed. It is noteworthy that the architecture of NLNNs can vary based on the implementation and task requirements [51]. Various adaptations and extensions of NLNNs have been proposed in the literature to address specific challenges and optimize performance in diverse domains.

The architecture of NLNNs is purposefully crafted to capture long-range dependencies and global context within an image. This design facilitates the extraction of meaningful features; as a result, this approach enhances the performance of neural networks in tasks like image detection, object recognition, and semantic extraction.

### 3.6. K-Means++

In object detection methodologies, achieving high-precision detection hinges on the utilization of suitable anchor boxes. Anchor boxes represent a predefined set of initial regions characterized by fixed dimensions and aspect ratios. The effectiveness of model training is contingent upon the alignment of predicted boundary boxes with actual boundary boxes, emphasizing the importance of anchor parameters. Specifically, the original YOLOv5 model necessitates customization of anchor parameters to cater to the requirements of specific datasets during the training process.

To address this, K-means clustering, recognized for its simplicity and efficiency, has been incorporated into the YOLOv5 model to derive the initial anchor boxes. However, the conventional K-means algorithm involves challenges related to the artificial setting of initial clustering centers, potentially yielding discernible differences in the final clustering output. One key limitation of the K-means algorithm is its reliance on specified inputs, such as the initial clustering centers and the predetermined number of clusters denoted as ‘k’. Determining the exact locations of clusters and choosing the initial cluster centers beforehand can be challenging and may impact the algorithm’s effectiveness.

In this study, the K-means++ algorithm was employed to obtain the initial set of anchor boxes (‘k’). The K-means++ algorithm addresses inherent issues in the original K-means algorithm by optimizing the selection of initial points. This optimization process, particularly beneficial for detecting small objects, significantly mitigates classification error rates associated with anchor box sizes.

### 3.7. SPPF+

The most recent version of YOLOv5 incorporates the spatial pyramid pooling fast (SPPF) as the conclusive module within the model’s backbone. This SPPF module consists of three layers of 5 × 5 MaxPool operations, wherein inputs undergo iterative processing. Subsequently, the output from these layers is concatenated before the execution of the CBS (Convolutional Block and Shuffle) operation. The spatial pyramid pooling fast (SPPF) technique leverages skip connections and maximal pooling to capture features at different scales. This method enhances the feature map’s representational quality by combining local and global characteristics. To weed out unimportant information and concentrate on key characteristics, maximum pooling, which extracts the maximum value from a collection of image regions using a rectangle mask, is utilized. However, it is noteworthy that while maximum pooling aids in reducing extraneous information, it may lead to the exclusion of less informative feature data.

This research endeavor elevates the notion of feature reuse by implementing a dense link construction inspired by DenseNet to enhance the spatial pyramid pooling fast (SPPF). Through this approach, we derive the SPPF module, strategically designed to mitigate the loss of feature information associated with maximum module pooling. The resultant SPPF+ module aptly preserves global information crucial for discerning fires impacting diminutive target forest areas.

### 3.8. Fine-Tuning, Transfer Learning, and Model Training

Insufficient training data can adversely impact the effectiveness and accuracy of deep learning (DL) tasks [52]. Nevertheless, transfer learning serves as a remedy by enabling models to achieve significant results without the need for extensive data. In this investigation, we embraced transfer learning and employed pre-trained weights derived from the COCO dataset to augment the performance of our model in the detection of various brain tumors. By capitalizing on the previously acquired features from COCO, our model gained essential image recognition capabilities crucial for the tumor detection process. Moreover, to further refine the pre-trained model, we implemented a technique known as fine-tuning. This process involved adjusting resource allocation to prevent memory depletion during both training and testing, thereby optimizing the overall performance of the model.

The initial phase of fine-tuning the model involved adjusting the default class numbers from 80 to 3, corresponding to the three types of brain tumors: glioma, meningioma, and pituitary. This modification became imperative due to the default number of classes in the COCO dataset being 80. As a result, the Conv filters, defined in the equation, needed to shift from the default value of 255 to 24. This adjustment was made considering that *C* represents the number of classes, five corresponds to the YOLO coordinates (which typically include coordinates for the bounding box and objectness score), and three denotes the number of various scaled bounding boxes *K* used in the YOLO algorithm.

*filters* = 3 ∗ (5 + *C*) (1)

To refine the YOLO-based model through fine-tuning, various hyperparameters, including batch size, subdivisions, learning rate, momentum, decay, and iterations, were specifically customized for this study, as detailed in Table 4. The fine-tuning process involved training the model with a batch size of 64, a subdivision of 8, and 6000 iterations. The learning rate, momentum, and decay values were optimized to align with the available resources, resulting in values of 0.00261, 0.9, and 0.0005, respectively. Furthermore, to monitor the training progress and obtain initial performance results, the weights were automatically serialized every 1000 iterations.

### 3.9. Evaluation Metrics

Following the completion of the training and testing phases, the subsequent step involved evaluating the model’s performance utilizing standardized metrics tailored for object detection. In this study, a threshold of 0.5 was applied to assess metrics, including Intersection over Union (IoU), Precision (PR), Recall (RE), and mean Average Precision (mAP). The calculation of these metrics was based on the identification of True Positives (TP), False Positives (FP), and False Negatives (FN) by the model. The evaluation process was conducted using a test set comprising 610 MRIs [53].

In the evaluation process, TP denotes the correctly detected tumor classes with accurate labels, FP signifies non-tumors that were incorrectly detected, and FN represents tumors that went undetected by the model. Notably, as the dataset did not encompass negative samples (MRIs without lesions or tumors), True Negatives were not factored into the assessment. Consequently, the F1-score was deemed a more appropriate metric for evaluating the harmonic mean between FNs and FPs in the context of an unbalanced dataset, offering a more robust evaluation than relying solely on accuracy [54].

(2) $${AP}_{C_{ij}}=\frac{1}{m}\sum_{j=1}^{m}{Precision}_{C_{ij}}$$

The mean Average Precision (mAP) is computed by averaging the values of Average Precision (AP) calculated for each category [55]. Utilizing mAP as the primary metric allows for the identification of the model that attains the most superior overall performance in the specific task of detecting brain tumors. The mathematical representation of the equation for calculating mAP is formally expressed as follows:

(3) $$mAP=\frac{1}{N}\sum_{i=1}^{N}{AP}_{i}.$$

The Intersection over Union (IoU) quantifies the degree of overlap between two bounding boxes. The IoU is calculated using the following equation, which determines the IoU by dividing the intersection area of the boxes by the area of their union.

(4) $$IoU=\frac{Area\;of\;Intersection}{Area\;of\;Union}$$

In the medical domain, metrics like Precision (PR), Recall (RC), and F1-Score play a crucial role in evaluating the accuracy of positive predictions among all detections, potential detections, and achieving a balance between PR and RE, respectively. Likewise, within the realm of Deep Learning (DL), these metrics are employed to assess a model’s performance and determine its reliability for a specific task. The calculations for these metrics are based on the equations below:

(5) $${PR}_{C_{ij}}=\frac{{TP}_{C_{ij}}}{{TP}_{C_{ij}}+{FP}_{C_{ij}}},$$

(6) $${RC}_{C_{ij}}=\frac{{TP}_{C_{ij}}}{{TP}_{C_{ij}}+{FN}_{C_{ij}}},$$

(7) $$F1-Score=\frac{2\;*\;(PR*RC)}{PR+RC}$$

These measurements offer important insights into how well the model can accurately predict positive outcomes, its sensitivity to detecting relevant instances, and the overall balance between precision and recall in addressing the complexities of medical and deep learning applications.

## 4. Experimental Results and Discussion

### 4.1. Overall Model Performance

**Figure 5:**
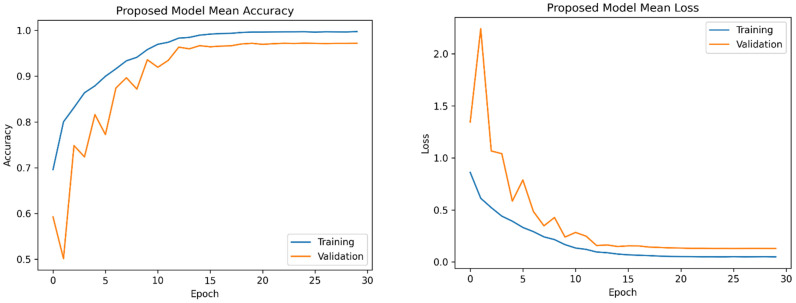
The mean accuracy and losses of the proposed model were assessed using 5-fold cross-validation. On the left side, you can observe the progression of mean accuracy across the training folds. On the right side, the corresponding trend in mean loss is depicted. These results show a consistent improvement in accuracy and a decrease in loss over the training folds, demonstrating effective model training.

In this section, we present an exposition on the outcomes derived from the training and evaluation processes applied to the fine-tuned YOLOv5 model using magnetic resonance imaging (MRI) images, accompanied by a thorough performance analysis. A series of preprocessing techniques and data augmentation methods were implemented to augment the dataset. The suggested model underwent training with varying hyperparameters in order to optimize its performance. The training procedure for the refined YOLOv5 model occurred on a personal computer equipped with Nvidia GeForce 1080Ti GPUs and 32 GB RAM. Figure 5 illustrates the average accuracy and losses incurred by the model proposed in this research study.

### 4.2. Comparison and Evaluation of the Proposed Method against State-of-the-art Techniques

**Figure 6:**
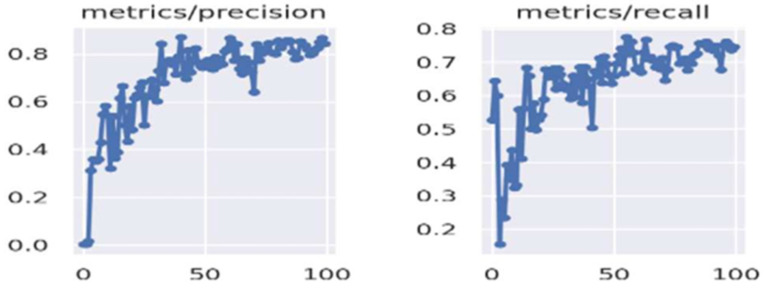
Precision and Recall metrics of brain tumor detection using improved YOLOv5.

The model proposed in this study, derived from the fine-tuned YOLOv5 architecture, underwent a comprehensive evaluation and comparison with contemporary methodologies for brain tumor detection, as outlined in [56]. In this section, we present a detailed analysis of its performance relative to these established techniques. The evaluation of the proposed model involved a meticulous examination utilizing benchmark datasets and established metrics for assessment. Performance metrics, including accuracy, precision, recall, and F1 score—commonly employed in the evaluation of object detection models—were utilized to gauge the effectiveness of the proposed model [57]. Figure 6 portrays the Precision and Recall metrics for brain tumor detection employing improved YOLOv5. Comparative analysis with existing state-of-the-art techniques revealed that the proposed model exhibited either competitive or superior performance, particularly in terms of overall accuracy and detection capabilities. The fine-tuning process, entailing the training of the YOLOv5 model specifically for brain tumor detection, significantly contributed to its enhanced performance in this specific task.

An inherent strength of the proposed model lies in its capacity to accurately and efficiently detect brain tumors within MRI images. Leveraging the advanced object detection capabilities embedded in the YOLOv5 architecture, the model excels in identifying brain tumor regions with a remarkable level of precision. This attribute proves pivotal for early detection and diagnosis, facilitating prompt medical intervention. Furthermore, the integration of YOLOv5 into the proposed model affords real-time or near-real-time performance, rendering it particularly suitable for applications demanding swift and efficient tumor detection, such as in clinical settings.

The comparative analysis of models trained using YOLOv5 and YOLOv5 in conjunction with NLNNs is delineated in Table 5, offering a comprehensive overview of the analysis process and its corresponding outcomes. The architectural design and optimizations incorporated into the model adeptly manage computational demands, effectively harnessing both CPU and GPU resources. Although the evaluation results indicate competent performance, further research and assessment employing diverse datasets and comparisons with other state-of-the-art techniques would contribute to a more nuanced understanding of the proposed YOLOv5-based model’s efficacy in brain tumor detection tasks and its potential advantages in this domain.

**Figure 7:**
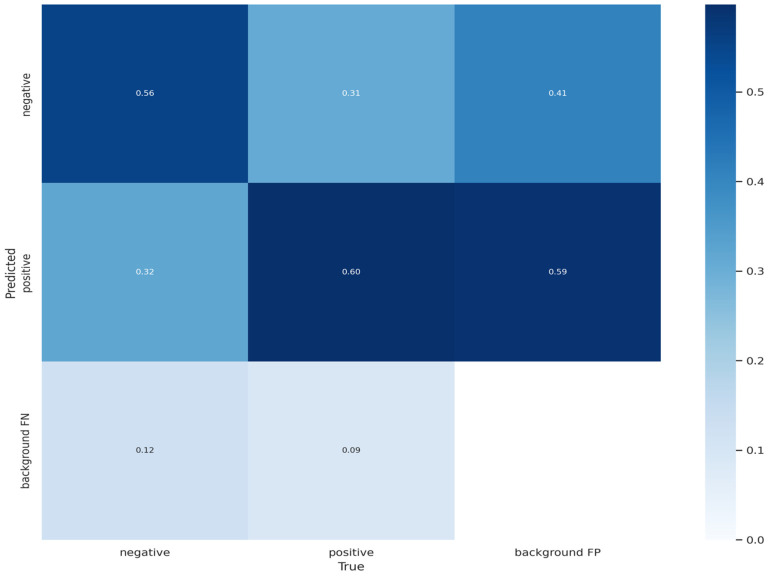
The result of confusion matrix for brain tumor detection.

This section furnishes a synopsis of the performance outcomes derived from the conducted experiments. It is imperative to underscore that those diverse models were trained, employing varying input sizes, to discern the most efficacious variant for addressing the specified problem. Figure 7 visually represents the confusion matrices pertaining to the proposed model, utilizing the testing data.

**Figure 8:**
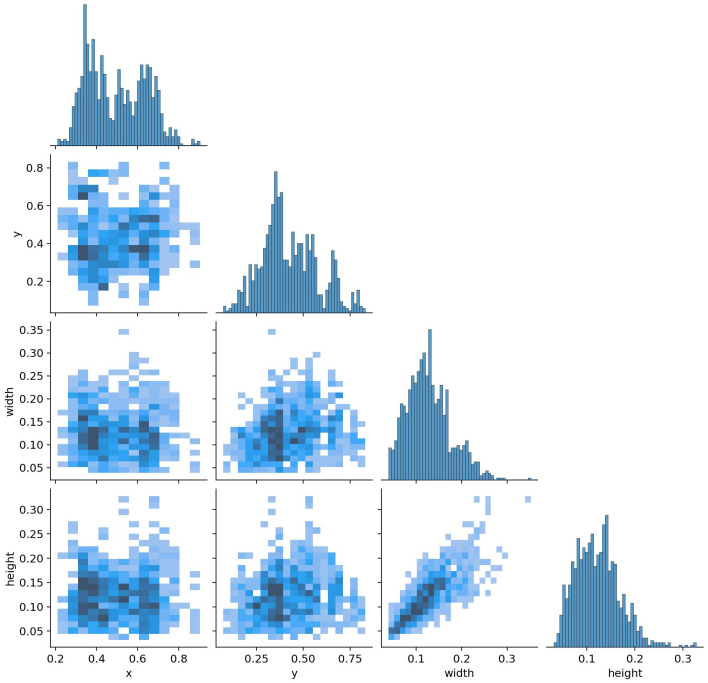
Labels correlogram of brain tumor dataset.

Figure 8 shows a label correlogram which is a graphical representation of brain tumor dataset and visualization. It is a visual tool that helps to display relationships and associations among labels or categories within a dataset.

The process of training the model by our proposed method consisted of 6000 epochs. The model spent 1.28 it/s per iteration in this process. It took a total of 2.1 h for 6000 iterations to train the model. We tried to use several factors to speed up this time spent. For example, we trained the model using a computer GPU device. This, in turn, accelerated the train time. The main reason for this increase in time is the large number of iterations. But our model has given a satisfactory result for each iteration process as we mentioned in the above sections.

The label correlogram typically consists of a grid where each cell represents the correlation or association between two labels or categories. The cells are filled with colors or patterns indicating the strength and direction of the relationship. This can be particularly useful when dealing with categorical data or variables, allowing for a quick overview of how different categories relate to each other.

**Figure 9:**
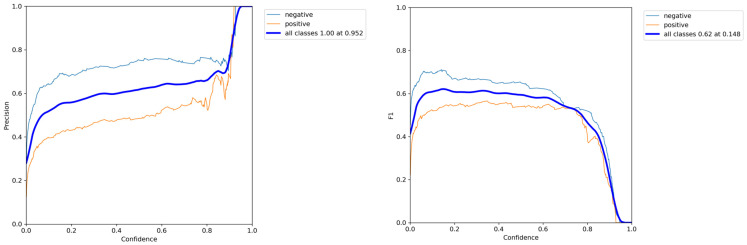
The results of precision and F1-score curve.

Based on the analysis of the results in Figure 9, it can be concluded that the proposed method produces satisfactory or favorable outcomes.

**Figure 10:**
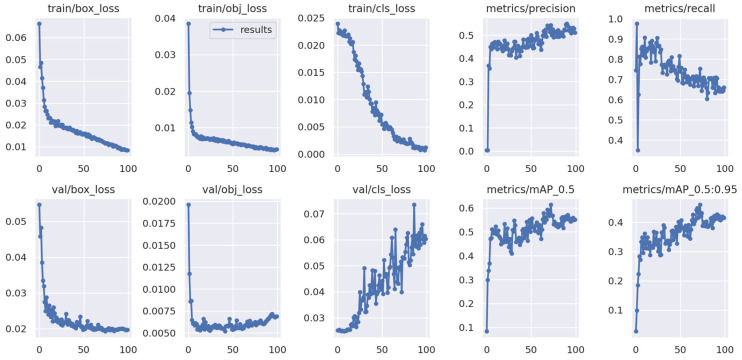
Results of training and validation set.

In the following Figure 10, we can see that the proposed YOLOv5 model performs well in training and validation set.

In Section 4, we conducted an ablation study comparing the enhanced fine-tuned YOLOv5 with the original YOLOv5 model. Table 5 and Table 6 display the results, offering insights into the effects of various attention mechanisms on model accuracy and information capture. Through a systematic exploration of NLNNs+, K-means, and spatial pyramid pooling fast+ (SPPF+) modules, illustrated in Figure 4, we discerned how these enhancements influence the performance of our YOLOv5-based brain tumor detection model. This experimental approach allowed us to gain a valuable understanding of each component’s contribution, thereby facilitating the refinement and optimization of an accurate brain tumor detection method.

**Figure 11:**
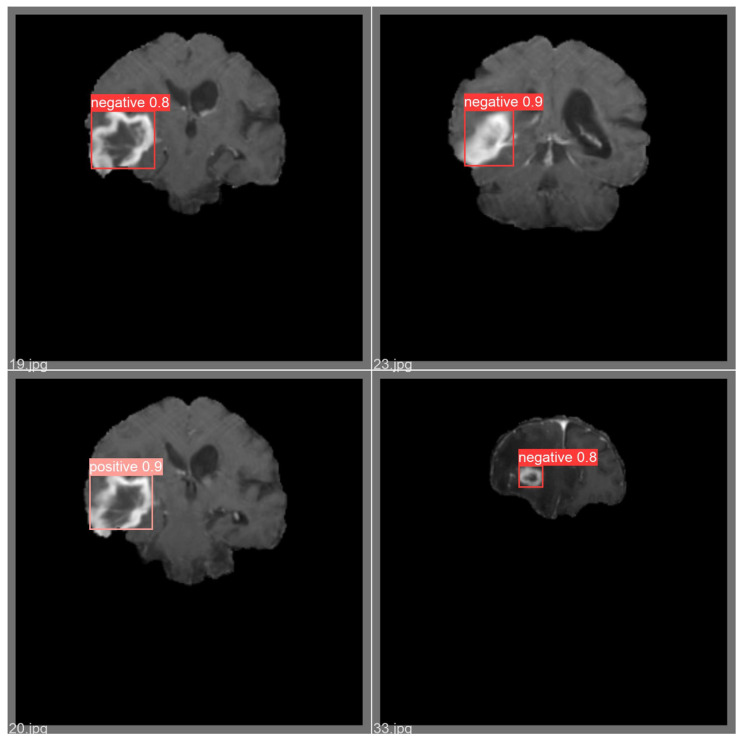

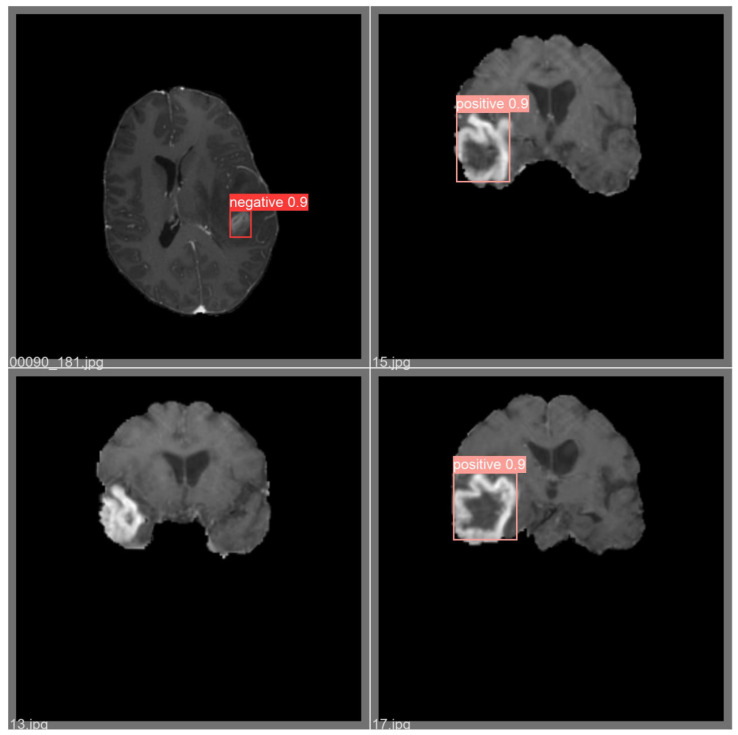
Example of qualitative result for brain tumor detection.

In addition, a qualitative assessment of the proposed brain tumor detection methodology was conducted. To achieve this objective, four images were randomly chosen from the test set of the brain tumor detection dataset. The qualitative outcomes of the refined YOLOv5 model for these selected images are depicted in Figure 11. It is noteworthy that these four images exhibit diverse sizes and contextual variations. As illustrated in Figure 11 the application of the proposed brain tumor detection approach using the enhanced YOLOv5 model demonstrated precise identification of brain tumors across varying sizes.

In our study, we addressed the issue of excess black area surrounding the brain region in the MRI images. To mitigate this effect, we performed a cropping process to remove the non-brain regions, specifically the black areas, while preserving the brain region of interest. This cropping step was undertaken to enhance the quality of the input data, ensuring that our brain tumor detection model focuses solely on the relevant brain structures, ultimately improving the accuracy and reliability of our findings. For these operations, contours were detected from the top, bottom, left, and right directions based on the presence of black regions.

In the experimental section, we used MRI images from an existing dataset. We are trying to detect small tumors as much as possible to show the effectiveness of the proposed method. Indeed, the proposed model may encounter challenges in accurately detecting small brain tumors, as deep learning models heavily rely on the training images for learning. To address this limitation and enhance the model’s performance, future improvements can be achieved through the creation of a dedicated dataset comprising small brain tumor images [65,66,67,68,69,70]. By assembling a comprehensive dataset specifically focused on small brain tumors, we can expose the model to a diverse array of such cases, enabling it to better discern the subtle characteristics and intricate patterns associated with these tumors. This process will facilitate the extraction of relevant features and enhance the model’s ability to detect small tumors more effectively. Through ongoing efforts to curate a representative and diverse dataset, we can iteratively train and refine the model, ultimately augmenting its sensitivity and specificity in detecting small brain tumors. Emphasizing such dataset curation and model enhancement endeavors will contribute significantly to the continued progress of deep learning applications in the field of brain tumor detection, ultimately benefiting patients and clinicians alike in the medical community [71,72,73].

## 5. Conclusions

This study highlights the efficacy of utilizing pre-trained and fine-tuned object detection models, specifically exemplified by the YOLOv5 model, for the accurate diagnosis of brain tumors from MRI images. In contrast to classification techniques, the proposed methodology excels in precisely localizing brain tumors within MRI scans, presenting specific classifications with reduced intricacy. Moreover, its compatibility with diverse platforms is notable due to its modest storage requirements and low computational overhead, distinguishing it from segmentation methods. Based on the experimental results and evaluation, it was concluded that the enhanced YOLOv5 model is robust and outperforms other methods in the precision and recall metrics, with 83.5% and 86%, respectively, on the brain tumor dataset. Comparative analysis with previous studies employing bounding box detection methodologies for meningioma, glioma, and pituitary brain tumors reveals superior precision in this work. Nonetheless, it is crucial to acknowledge inherent limitations associated with the bounding box detection approach utilized herein. The use of bounding boxes may compromise the meticulous delineation of tumor boundaries when contrasted with segmentation techniques.

We acknowledge the imperative need for additional investigation and comprehensive testing to thoroughly validate the efficacy of our proposed method. Our study advances this field by utilizing five different convolutional models and transfer learning architectures. Brain tumor diagnosis via medical imaging is still a major area of research attention. Still, there is a lot of potential in this subject for more research and development. Both patients and medical professionals dealing with the difficulties of treating brain malignancies stand to gain from the further development of brain tumor detection systems through ongoing research. We can increase diagnostic capabilities and, eventually, patient outcomes by improving detecting technologies and deepening our understanding of this field [74,75].

The future direction of our research involves conducting comprehensive performance evaluations of the proposed method using a larger dataset. This will help us assess its ability to distinguish between various types of brain lesions. While the current dataset serves as an initial step for brain tumor detection, future studies should aim to incorporate a more diverse and clinically relevant range of brain lesions, addressing the complexities found in real-world diagnostic scenarios. This approach will provide a more robust and applicable understanding of the model’s performance across different clinical situations [76]. Additionally, creating synthetic images with small lesions, guided by the expertise of medical professionals, can enhance the dataset and ensure the model encounters cases that may not be present in real data. Furthermore, combining predictions from multiple detection models, each trained on different subsets of data, can improve overall detection performance, particularly when dealing with diverse lesion sizes [77].

## Figures and Tables

**Table 1:** An overview of various algorithms used in the diagnosis of brain tumors.

Author	Model	Approach	Performance	Year
Cheng Almahfud et al. Pereira S	Novel-segmentation model K-means and FCM-clustering CNN-based	Segmentation Segmentation Segmentation	mAP of 94.68% Accuracy of 91.94% DSC 88%	2016 2018 2016
Bhanothu	Faster R-CNN	Detection	mAP 77.60%	2020
Swati	VGG19	Classification	Accuracy of 94.82%	2019
Deepak	GoogleNet	Classification	Accuracy of 98%	2019
Rehman	AlexNet, GoogleNet, VGG16	Classification	Accuracies of 97.39%, 98.04%, and 98.69%, respectively	2019
Sultan	Custom-CNN	Classification	Accuracy of 98.7%	2019
Noreen	DenseNet201 and InceptionV3	Classification	Accuracies of 99.34% and 99.51%	2020

**Table 2:** The MRI brain tumor dataset specifications.

Class	Coronal	Axial	Sagittal	Total
Meningioma	232	208	268	708
Glioma	493	494	439	1426
Pituitary	321	291	318	930
Total	1046	993	1025	3064

**Table 3:** Distribution of datasets for testing and training.

Class	Train	Test	Total
Meningioma	565	140	705
Glioma	1140	280	1420
Pituitary	740	180	920
Total	2450	610	3060

**Table 4:** Model fine-tuning using the hyperparameter setup.

Hyperparameter	Value
Batch Size	64
Subdivisions	8
Learning Rate	0.00001
Warmup Epochs	3.0
Box	0.05
IOU Threshold	0.20
Momentum	0.9
Decay	0.0005
Iterations	6000

**Table 5:** Comparison analysis of YOLOv5 and improved YOLOv5 with NLNNs.

Model	Precision	Recall	mAP
YOLOv5	81.9	83	87
Improved YOLOv5	83.5	86	85.2

**Table 6:** Comparative analysis of proposed work with previous works.

Contribution	Model	Approach	Accuracy (%)	Dataset
Soheila Saeedi et al. [58]	Elementary features-model-based	Detection	96.47%	Brain tumor classification (MRI): four classes neural network
Sarmad Maqsood et al. [59]	MobileNetV2	Segmentation	97.47%	T1-weighted contrast-enhanced MRI
Shah Hussain et al. [60]	U-net ETISTP Model	Segmentation	96%	T1-weighted contrast-enhanced MRI
Ejaz Ul Haq et al. [61]	CNN classifier	Classification	97.3%	T1-weighted contrast-enhanced MRI
S. Patil et al. [62]	SCNN classifier/VGG16	Classification	97.7%	MRI dataset
Talukder et al. [63]	DL (ResNet50V2)	Classification	99.6%	Brain tumor classification (MRI): three classes
Woźniak et al. [64]	CNN classifier	Classification	95.7%	CT brain tumor classification
Abdusalomov et al. [65]	YOLO7	Classification	99.5%	MRI scan images (kaggle): four classes
	InceptionV3			

## Data Availability

Data are contained within the article.
